# Assessment of potential exposure to As, Cd, Pb and Zn in vegetable garden soils and vegetables in a mining region

**DOI:** 10.1038/s41598-022-17461-z

**Published:** 2022-08-05

**Authors:** Kateřina Vejvodová, Christopher Ash, Julie Dajčl, Václav Tejnecký, Hana Johanis, Marko Spasić, Filip Polák, Lukáš Praus, Luboš Borůvka, Ondřej Drábek

**Affiliations:** 1 Department of Soil Science and Soil Protection, Faculty of Agrobiology, Food and Natural Resources, Czech University of Life Sciences, Prague, Czech Republic; 2 Laboratory of Environmental Chemistry, Faculty of Agrobiology, Food and Natural Resources, Czech University of Life Sciences, Prague, Czech Republic

**Keywords:** Environmental chemistry, Environmental sciences, Environmental chemistry

## Abstract

Mining and smelting activities can contaminate soils and affect farming due to high emissions and input of potentially toxic elements (PTE) into the environment. Soils (sampled from two depths) and market vegetables from vegetable gardens located within the vicinity of unconfined slag deposits from decades of mining and smelting activities in Kutná Hora, Czechia were assessed to determine to what extent they pose a health hazard to communities that use these gardens. Pseudo-total As concentrations in the soils exceeded background levels (4.5 mg kg^−1^) 1.9–93 times, with higher concentrations in the deeper layer. The pseudo-total concentrations of PTE in soils ranked in the order As > Zn > Cd > Pb. Phyto-available concentrations of PTE in soils were relatively low, compared to pseudo-total concentrations. Concentration of As, Cd, Pb and Zn in the vegetables exceeded guideline values, with the highest concentrations found in the fruits of cucumber, peppers, and zucchini. Despite low phyto-available PTE concentrations in soils, all the PTE concentrations in the vegetables surpassed the guidelines set by the Czech Ministry of Health and EU directive, indicating a health hazard to consumers.

## Introduction

The Czech town of Kutná Hora (KH) and its surrounding landscape represents one of the oldest and most significant Bohemian mining regions, dating back to the thirteenth century. At its peak, annual silver production stood at 5–6 tons^1,2^. The town’s intensive mining history is evident from the presence of a network of mineshafts, excavated overburden, and unconfined smelter slag deposits. Drahota et al.^3^ studied the mine wastes, urban soils and road dust and confirmed that As was the main and the most important contaminant in urban soils (up to 2900 mg kg^−1^) followed by Ag, Cu, Pb and Zn. Ash et al.^4^ studied the potentially toxic element (PTE) concentrations in soils at a slag deposit alongside the Vrchlice River in surrounding area of KH. Despite determining very high loadings of PTE in soil samples, phyto-available contents (using CaCl_2_ and Mehlich extractions) were generally low, which concurs with findings of Száková et al.^5^ and Tremlová et al.^6^. Horák and Hejcman^7^ used available data on PTE distributions in KH to group PTE based on their likely sources; those uninfluenced by mining activities (Be, Co, Cr, Hg, and V), originating from smelting processes (As, Cu, Pb and Zn), and originating from mining (As and Cd). Large-scale ore exploitation which begun in the early fifteenth century was due to the development of new smelting technology^2^. This technology used some sulphides as additives for silver ore smelting, which contained small amounts of arsenopyrite (AsFeS), sphalerite (ZnS) and galena (PbS), with Ag in KH being bounded to arsenopyrite, and therefore being a source of As in the smelting processes. Moreover, some As minerals (alacránite (As_8_S_9_), allargentum (Ag1–xSb_x_) and arsenopyrite) also enter the smelting process^8^. Thus, the smelting activities became a massive source of contamination for the entire surroundings^3,4,8^. Now covered by forest, there are numerous sinkholes from mineshaft collapse, and past excavations shape the landscape here. Slag fragments from the smelting litter the region, as it was either dumped to form unconfined heaps, or used in road construction, and even applied to fields as a primitive fertilizer (source of Ca and Mg). Unconfined heaps that were rich in arsenopyrite and Fe-sulphides were left for almost 500 years, leaving them exposed to weathering, breaking the sulphide into weathered As-, Fe-, and S-rich waste materials^8^.

Unconfined deposits of slag are exposed to natural agents, and therefore weather over time to smaller and lighter particles that can be transported as dust during strong winds, furthermore, historical processing of the mined material have resulted in widespread pollution of the Kutná Hora region. Many of its residents are unaware of the contamination extent. This study aimed at determining whether soil and edible plant parts from vegetable gardens surrounding the area of historic Ag/Cu mining represent a likely health hazard due to exposure to PTE sources. The potential level of exposure through vegetable and fruit consumption was quantified by comparing observed PTE concentrations to national safety guideline values (Decree No. 53/2002)^9^. Soil samples were compared to soils from vegetable gardens in an area without any known pollution source.

## Materials and methods

**Figure 1:**
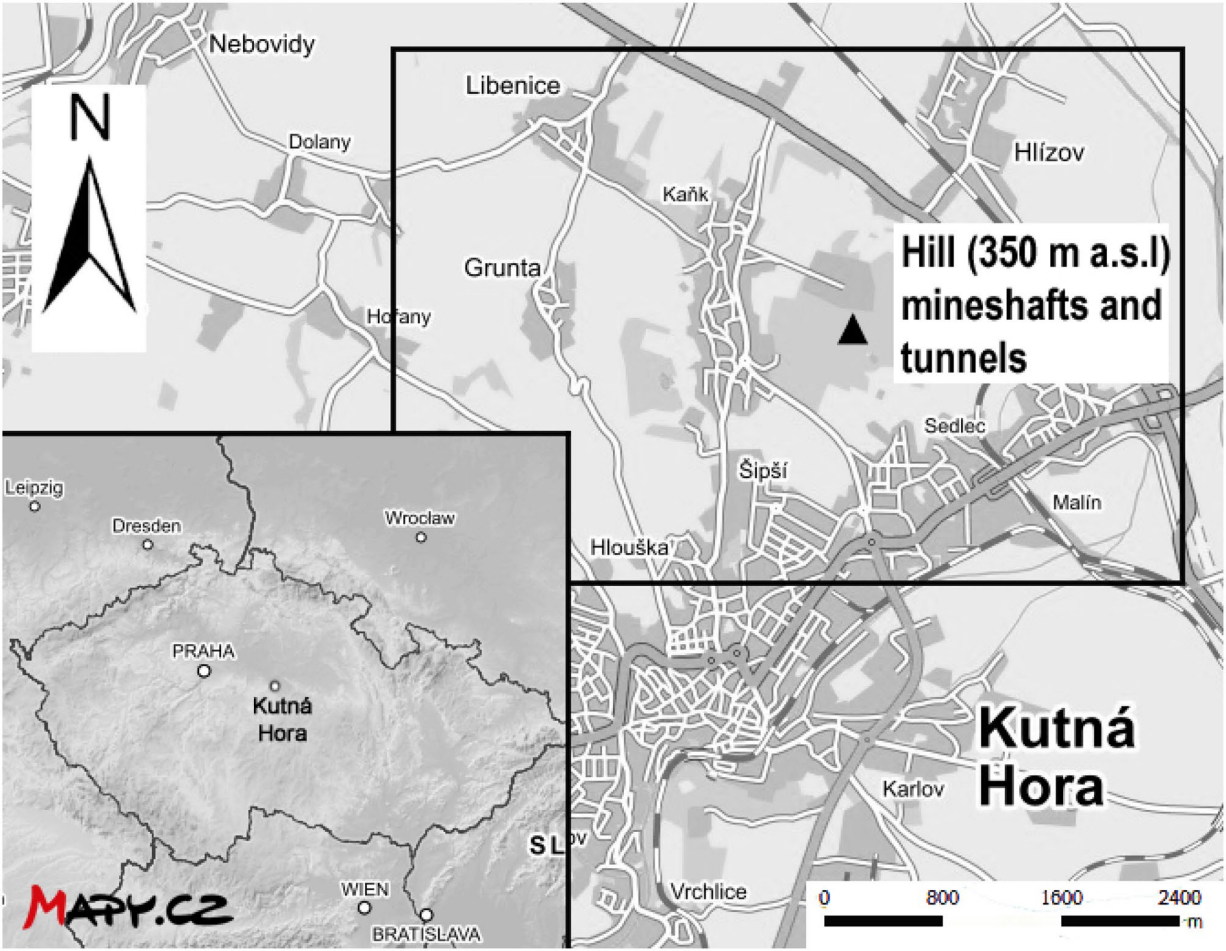
Map showing the region of Kutná Hora, Czechia in which extensive mining activities took place (source: mapy.cz; processed with Adobe Photoshop Elements 7.0). Samples were collected from a number of unspecified vegetable gardens to the north of the town.

Soil and plant samples were collected with permission from private vegetable growers located in the vicinity of Kutná Hora (Czechia, altitude 255 m a.s.l; latitude 49° 56ʹ N; longitude 15° 16ʹ E; mean annual rainfall 486 mm, temp. 9 °C.); a map of the general area with some key features is given in Fig. 1. The geological sub-surface of KH consists of metamorphic rocks, mainly different types of gneisses, and mica-schists, quartzitic erlan/calc-silicate rocks, and migmatites^10^. Their top walls are made of platform sediments, sandstones and organodetrital limestones/coquina of the Bohemian Cretaceous Basin, originating from the Cretaceous age^7^.

### Soil and plant sampling

**Figure 2:**
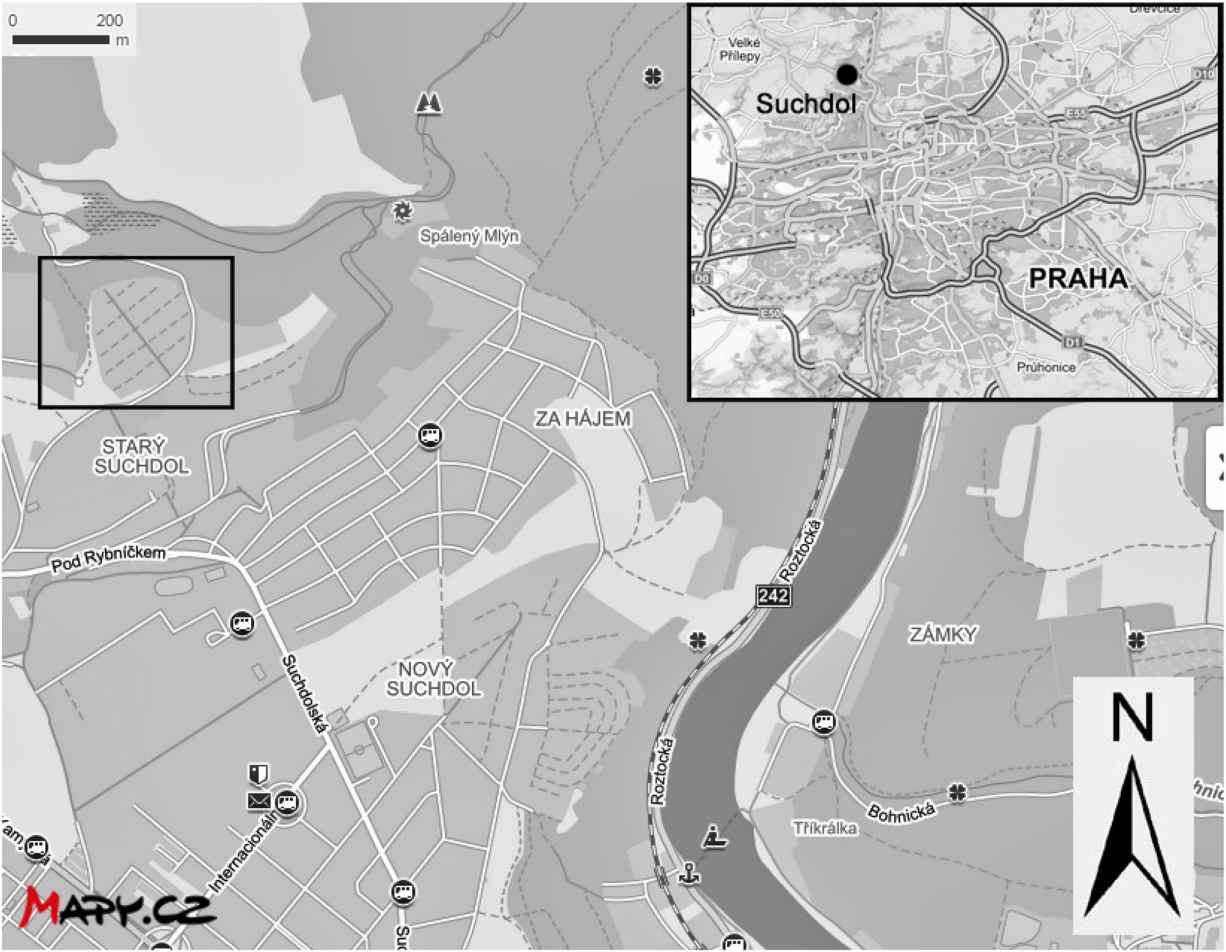
Map showing the location of the growers’ association plots in Suchdol, Czechia from where control samples were collected (source: mapy.cz; processed with Adobe Photoshop Elements 7.0). Altitude 260 m a.s.l; latitude 50° 13ʹ N; longitude 14° 37ʹ E; mean annual rainfall 554 mm, temp. 8.5 °C.

One hundred and one cores were drilled in total (around 3 cores per plot). Plots chosen for sampling were random as the collection of soil depended on voluntary participation of individual plot owners. The sampling design was created as not to disturb the plots during vegetation period. From each core, soil was collected from 0 to 15 cm, and 15 to 30 cm depth; these were labelled as A and B, respectively. Samples were collected from two depths, to observe if there was PTE mobility. Samples were collected during the growing season within cultivated soil beds, from soils where only the top 20 cm undergo turbation. For a control against background sources of potential agricultural related inputs of PTE (e.g., fertilizer and pesticide applications), 45 cores were drilled in total from 15 plots at a growers’ association in Suchdol, Prague. Soil from A (0–15 cm) and B (15–30 cm) layers was collected. This area is mostly residential, with no industry and no significant polluting sources (Fig. 2). Plant samples were collected from 12 different gardens that included 8 different fruit and vegetables. These were apples (*Malus domestica* L. ‘Spartan’), tomatoes (*Solanum lycopersicum* L. ‘Start S F1’), cucumbers (*Cucumis sativus* L. ‘Admira F1’), onions (*Allium cepa* L.), garlic (*Allium sativum* L.), zucchini (*Cucurbita pepo* L. ‘Nefertiti’), potatoes (*Solanum tuberosum* L. ‘Karin’) and peppers (*Capsicum annuum* L. ‘Amy’. Only the edible plant parts were analysed. Fruit and vegetable samples that were most predominant in gardens were potatoes, peppers, tomatoes, and cucumbers.

### Sample processing

Soil samples from both the KH gardens and the control site were oven dried (30 °C) and analysed in the same way. Soil samples were sieved to < 2 mm. The edible plant samples were thoroughly cleaned, frozen, lyophilized (freeze drier, between − 60 °C and − 80 °C) and homogenised and crushed in a mill prior to analysis. Most samples were left to be analysed as they were, however, garlic and onions were peeled, so only the parts that are actually consumed by households were analysed.

Soil active and exchangeable pH was determined by preparing a 1:5 soil:liquid (w:v) ratio using deionised water (pH_H2O_) or 2 M KCl solution (pH_KCl_), the samples were agitated for 5 min and left to settle for 1 h (H_2_O) and 24 h (KCl), then measured with a Denver Instrument UB-5 pH meter. Content of soil oxidisable carbon was measured by a modified Tyurin’s titration method; samples were heated with potassium dichromate in sulphuric acid and were then back-titrated with Mohr’s salt^11^. A method to approximate the “pseudototal” PTE content in soils was used for comparison against Czech background soil values^12^. The pseudototal extraction does not include metals fixed within the minerals (e.g., those bound in the silicates). The following methodologies were adapted from^13^. Five grams of soil was shaken with 50 mL of 2 M HNO_3_ for 6 h on an orbital shaker. The mixture was then centrifuged, and the supernatant filtered (nylon 0.45 µm syringe filter). The phyto-available portion of PTE in soil was determined by shaking 10 g of soil with 40 mL of 1 M NH_4_NO_3_ for 2 h, followed by filtering through filter paper (Filtrak 390, Niederschlag, Germany (DIN 53,137)). Total concentration of PTE in plant samples were determined by reacting 0.5 g of plant dry samples with 65% HNO_3_ overnight and then heated at 160 °C for 2 h.

Potentially toxic elements in all soil extract solutions were analysed using ICP-OES iCAP 7000 (Thermo Fisher Scientific, Waltham, MA, USA); limit of detection (LD) was calculated according to the equation: LD = 3.29 σ0 (σ0 is blank sample standard deviation). Samples and standards were matrix matched. Procedural blanks were included in the analyses.

Potentially toxic elements in plant digestates were analysed using ICP-MS (single quadrupole inductively coupled plasma- mass spectrometry, Agilent 7700x, Agilent Technologies Inc., USA). The ICP-MS was fitted with a micro-concentric nebulizer and quartz Scott-type spray chamber. The analysis was performed in the No Gas (^111^Cd and ^206^Pb) and He mode (^63^Cu, ^66^Zn, and ^75^As) using an external calibration and ^72^Ge, ^103^Rh and ^175^Lu as internal standards. Two certified reference materials (CRM) were included in the whole procedure for quality assurance, namely Tea leaves (INCT-TL-1 and NCS DC73351). The limits of detection (LDs) for the whole analytical procedure (PTE in plants) were 0.036 mg Cu kg^−1^, 0.39 mg Zn kg^−1^, 0.008 mg As kg^−1^, 0.0012 mg Cd kg^−1^, and 0.0032 mg Pb kg^−1^. Experimental research and field studies on plants complies with relevant institutional, national, and international guidelines and legislation.

Data handling and basic statistical (correlations) applications were made in Microsoft Excel. Independent t-tests were performed using Statistica 13 software (StatSoft, Inc).

### Consent to participate

The authors provided consent to participate in this study.

### Consent to publish

The authors provided consent to publish this study once accepted.

## Results and discussion

### Basic soil properties

The oxidisable carbon (Cox) measured in the soil samples ranged within the common values for tilled agricultural soil, which is generally < 5% (Table 1)^14^. A weak positive correlation was observed between HNO_3_ extractable Cu and Cox (P ≤ 0.05). Lead, Zn and Cd also showed positive correlation but to only a slight extent, whereas correlation between As and Cox was negative in both soil layers. The slight to no correlation between Cox and the extractable soil PTE proves that if there was any additional organic matter (OM) input to the soil, it did not significantly add to the topsoil contamination, albeit available PTE released by OM mineralization may be either taken up by roots of the following cultures or leached. A weak positive correlation between Cu and Cox can be expected due to the Cu affinity for organic matter. Soil pH was neutral for the majority of samples, with some ranging to the moderately alkaline spectrum^15^. No significant relationships were determined between soil pH and the phyto-available PTE (as a percentage of HNO_3_ extract).

**Table 1:** Basic properties of Kutná Hora soil samples.

	Cox %		pH_H2O_		pH_KCl_	
	A	B	A	B	A	B
Min	0.15	0.58	6.62	6.78	6.29	6.45
Median	1.81	2.08	7.40	7.45	7.10	7.11
Max	5.61	5.03	8.02	8.05	7.60	7.48
Mean	2.04	2.09	7.40	7.45	7.10	7.11
s.d	1.05	0.86	0.27	0.25	0.24	0.20

*Cox* oxidisable carbon, *s.d.* standard deviation, *A* upper layer (0–15 cm), *B* lower layer (15–30 cm).

No significant differences (P =  < 0.05) between layers; no. of soil samples- 101.

### Potentially toxic elements in soil

The highest pseudo-total soil PTE concentrations were for As, followed by Zn, Cd and Pb. The sample containing the lowest As concentration (min.) exceeded the soil background level (SBL)^12^ 1.9 times (Table 2). The SBL allows for the comparison between contaminated soils and background levels of PTE in soils. In the most contaminated sample, As exceeded the SBL 93 times. Regarding differences between soil layers, a slightly higher pseudo-total As concentration was found in the deeper B layer (possibly due to leaching); HNO_3_ extractable median of 54.5 mg kg^−1^ as opposed to 49.1 mg kg^−1^ in the A layer. However, the greatest maximum content of As was observed in the A layer (418 mg kg^−1^). Horák and Hejcman^7^ performed a large-scale characterization of pollution levels in the region north of KH. Interpolations of PTE showed that As was frequently found in the range of hundreds to thousands of mg kg^−1^. The large number of dumps of waste rock and slag in the area surrounding the gardens contain not only primary minerals of As, but also secondary minerals. Secondary As minerals such as bukovskyite (Fe^3+^_2_(AsO_4_)(SO_4_)(OH)), pitticite (Fe^3+^_20_(AsO_4_,PO_4_,SO_4_)_13_(OH)_24_·9H_2_O), and scorodite (Fe^3+^(AsO_4_)·2H_2_O) were created by weathering of arsenopyrite, and also zykaite (Fe^3+^_4_(AsO_4_)_3_(SO_4_)(OH)·15H_2_O), kankite (Fe^3+^(AsO_4_)·3.5H_2_O), and parascorodite (Fe^3+^(AsO_4_)·2H_2_O)^16–19^. Arsenic is firmly bound to oxides of Fe/Al in the form of arsenite(III) or arsenate(V)^8^, and so can be considered largely immobile in mineral type soils; this is reflected in the relatively low phyto-available portion of this element in the studied soils (mean = 2.1% and 2.4% in A and B layers respectively). However, when exposed to soil solutions containing organic anions in the dissolved organic carbon (DOC), e.g., organic acids such as oxalic acid, citric acid, and malic acid, research (including research done by Ash et al.^20^) has shown that As can be released into solution by various mechanisms, including the complete dissolution of the mineral oxide to which As was bound^20,21^. Therefore, the addition of organic residues and manures to soil is likely to enhance the mobility of As and its potential uptake by plants. At the same time however, with sufficient irrigation the released As can be leached to greater soil depth, thus eliminating the pathway of As exposure by inhalation or ingestion of contaminated soil at the surface. Another option could involve the use of Fe-oxides in order to sorb mobile As^22^. Despite its generally low relative availability, As was the most phyto-available element compared to the other studied PTE, although control soils had only a slightly lower percentage of availability. Congruent to observations by Xu and Thornton^23^, who studied As-contaminated gardens at a mining area in southern England, the phyto-available content correlates with the total As content (R = 0.80 and 0.81 for A and B respectively).

**Table 2:** Potentially toxic elements in Kutná Hora soil samples.

	As						Cd					
	HNO_3_ (mg kg^−1^)		Available (%)		Factor for exceeding SBL		HNO_3_ (mg kg^−1^)		Available (%)		Factor for exceeding SBL*	
	A	B	A	B	A	B	A	B	A	B	A	B
Min	8.7	15.2	0.43	0.38	1.94	3.38	0.27	0.35	0.61	0.77	0.27	0.35
Median	49.1	54.5	2.09	2.25	10.9	12.1	0.80	0.81	1.50	1.31	0.80	0.81
Max	419	408	5.73	5.99	93.0	90.6	3.01	3.27	4.78	2.58	3.01	3.27
Mean	68.7	81.9	*2.12*	*2.42*	15.3	18.2	1.07	1.06	1.65	1.38	1.08	1.06
(c) Mean	*3.24	*3.22	*1.54	*1.27	0.72	0.72	*0.39	*0.37	*0.56	*0.44	0.39	0.37

	Pb						Zn					
	HNO_3_ (mg kg^−1^)		Available (%)		Factor for exceeding SBL*		HNO_3_ (mg kg^−1^)		Available (%)		Factor for exceeding SBL*	
	A	B	A	B	A	B	A	B	A	B	A	B
Min	19.6	26.2	0.001	0.001	0.28	0.37	32.3	42.1	0.10	0.08	0.32	0.42
Median	56.4	56.8	0.17	0.12	0.81	0.81	145	137	0.26	0.38	1.45	1.37
Max	113	178	9.44	2.10	1.62	2.55	597	759	2.31	2.52	5.98	7.59
Mean	60.0	64.2	*0.65*	*0.23*	0.86	0.92	165	171	*0.39*	*0.45*	1.65	1.72
(c) Mean	35.6	*32.2	*0.15	*0.09	0.51	0.46	*87.7	*71.4	*0.69	0.54	0.88	0.71

*SBL* soil background level, according to Beneš^12^ (SBL = As 4.5 mg kg^−1^, Cd 1.00 mg kg^−1^, Pb 70 mg kg^−1^, Zn 100 mg kg^−1^).

*Significant difference between KH and control (c) means at p ≤ 0.001 (independent t-test), Italic cells represent significant differences between layers A and B at p ≤ 0.05; no. of soil samples- 101.

Regarding Cd, more than a quarter of the data were in excess of the 1 mg kg^−1^ SBL limit. Enrichment with Cd in the KH soils is particularly evident when compared to the control soils. Cadmium is a metal that is characterised by generally higher mobility than other metals with similar valence, such as Cu, Pb, and Zn, which are associated with binding to organic matter carbonates and clays. Higher mobility of Cd usually translates into enhanced plant uptake but can also mean greater vertical leaching; in this case, little difference in total Cd contents between A and B layers was observed.

For Pb, concentrations exceeding the SBL were detected in approximately one quarter of the samples from both layers. However, the maximum pseudo-total Pb concentration was observed in deeper (15–30 cm) soil samples; this may reflect the smelting practices that took place in past centuries. Lead sulphides were added to the smelter to decrease the melting temperature of silver^24^; because smelting activities ceased long before the establishment of the vegetable gardens, it is likely that the most enriched soils have been buried by imported topsoil or newly developed surface soil layers. Lead isotope analysis would be necessary to confirm the Pb source. Independent t-test confirms the higher content of Pb in the B layer samples; nevertheless, both layers A and B contained considerably more Pb than in the control soil.

Besides As, Zn was the only PTE whose median concentration in KH soils was above the SBL. However, while excess Zn is phytotoxic, it is generally considered relatively nontoxic for animal and humans, and concentrations must be highly excessive for symptoms of toxicity to manifest in humans^25^. Furthermore, Zn is a micronutrient element in plants, and so concentrations at or near baseline or recommended guideline levels are not a concern^26^.

### Potentially toxic elements in plants

The plant samples (Table 3) were contaminated with higher concentrations of As, Cd, Pb and Zn than the allowable quantity (AQ) and maximum allowable quantity (MAQ) set by the Ministry of Health in the Czech Republic (Decree No. 53/2002)^9^. The plants samples also exceeded the maximum permitted concentrations of Cd (0.02–0.1 mg kg^−1^) and Pb (0.1 mg kg^−1^) set by the EU directive (Decree No 1881/2006)^27^.

**Table 3:** PTE concentrations in fruits and vegetables (mg kg^−1^ dry matter).

	Apple	Tomato	Cucumber	Onion	Garlic	Zucchini	Potato	Pepper
n value	2	5	3	3	2	1	4	6
**As**								
Min	0.46	0.15	4.24	1.49	2.90	0.63	0.39	0.48
Max	0.59	0.59	5.09	3.01	3.73	0.63	1.04	1.22
Mean	0.53	0.39	4.72	2.11	3.32	0.63	0.65	0.81
s.d	0.05	0.14	0.34	0.61	0.40	0	0.24	0.28
MAQ*	0.5	0.5	0.5	0.5	0.5	0.5	0.3	0.5
No. of samples exceeding MAQ	2	1	3	3	2	1	4	5
**Cd**								
Min	0.01	0.06	0.02	0.08	0.17	0.07	0.05	0.15
Max	0.01	0.26	0.05	0.13	0.23	0.08	0.30	0.68
Mean	0.01	0.15	0.03	0.10	0.20	0.07	0.12	0.37
s.d	0	0.06	0.01	0.02	0.02	0	0.07	0.21
MAQ*	0.05	0.1	0.1	0.1	0.1	0.1	0.1	0.1
No. of samples exceeding MAQ	0	4	0	2	2	0	3	6
**Pb**								
Min	0.87	0.57	0.43	0.89	0.52	1.32	0.66	0.60
Max	1.14	2.06	1.01	1.76	1.51	1.36	3.65	4.42
Mean	1.05	1.05	0.69	1.41	0.95	1.34	1.42	1.91
s.d	0.10	0.50	0.23	0.30	0.38	0.02	0.93	1.15
MAQ*	0.1	0.1	0.1	0.1	0.1	0.1	0.1	0.1
No. of samples exceeding MAQ	2	5	3	3	2	2	4	6
**Zn**								
Min	11.83	43.02	84.21	54.85	66.45	145.21	31.36	73.35
Max	22.13	65.95	97.42	72.90	117.01	152.46	73.56	108.27
Mean	16.44	56.22	91.09	64.05	86.47	148.83	49.60	88.76
s.d	3.84	7.50	4.09	6.74	20.54	3.62	12.07	11.88
AQ*	10	25	25	25	25	10	25	25
No. of samples exceeding MAQ	2	5	3	3	2	2	4	6

*MAQ* maximum allowable quantity (Decree of the Ministry of Health, CZ), *AQ* allowable quantity, *n value* number of samples, *s.d.* standard deviation.

Higher As concentrations occurred in cucumbers, onions, garlic, potato tubers, and peppers (max values reaching 5.09, 3.01, 3.73, 1.04 and 1.22 mg kg^−1^, respectively). Higher As concentrations in some plant parts could be explained by fractions of bioavailable As in soils, deposition of dusts on plants (that may contaminate the stomatal chambers) with above-ground edible biomass, longer planting periods and different garden plots and soils in the area^26^. Cadmium concentrations in edible plant parts were highest in several of the potato tubers and pepper plants, reaching concentrations up to 0.30 and 0.68 mg kg^−1^, respectively. Cadmium can be observed to being efficiently stored by root and leaf systems, depicting the bioavailability of Cd in soils (up to 5%), indicating a relationship between Cd in plants and Cd in the growth medium^26^.

Several factors that affect the concentrations of Pb in a plant are pollution and accumulation abilities of plants, with atmospheric deposition of Pb on above ground biomass being an important source of Pb contamination in plants^26,28^. The plant samples with the highest Pb concentrations were peppers, potato tubers, and tomatoes (max concentrations of 4.42, 3.65 and 2.06 mg kg^−1^, respectively).

Soluble Zn is readily available for plant uptake, however, rate of uptake is controlled by plant species and cultivars^26^. With regards to our results, Zn concentrations in the plants were up to 15 times higher than the AQ in the case of zucchini (Table 3). The high Zn concentrations in the edible plant parts correlated to the high concentrations in the soils, reaching up to 759 mg kg^−1^ in some samples.

Jolly et al.^29^ investigated transfer factors of PTE into different vegetables that were grown on soil with elevated PTE concentrations. They also observed a relative abundance of As, Cd, Pb and Zn in the edible parts of plants, with highest concentrations in Amaranthus and elevated concentrations also in tomatoes, radish, spinach and beans. Tremlová et al.^6^ found As concentrations ranging from 1.6 to 64 mg kg^−1^ in dried plant edible tissues grown on contaminated KH soils with limited plant available As in soils with highest concentrations in parsnip and black radish and lowest concentrations in savoy cabbage and lettuce. The study by Tremlová et al.^6^ presents results similar to this study, where we found low plant available As in soils, however, plant samples still surpassed As guideline values. Another study by Tremlová et al.^30^ found both low and high As concentrations in different plant species ranging from 0.02 to 39.30 mg kg^−1^ with arsenite and arsenate being the predominate As compounds. A study conducted by Králová et al.^31^ on soils contaminated by mining activities in KH showed low plant available concentrations for As and Pb (not exceeding 0.5% of pseudototal) and relatively high plant available concentrations for Cd and Zn (47 and 60%, respectively). In the aboveground biomass of the plants studied by Králová et al.^31^, low As concentrations were found (ranging from 0.36 to 3.64 mg kg^−1^) in the plant species, indicating a low translocation rate. In our study, As concentrations in our plant samples were up to 5.09 mg kg^−1^, therefore concentrations were much lower than results presented by Tremlová et al.^6,30^ but similar to Králová et al.^31^. Cadmium in the study by Králová et al.^31^, was more readily translocated in the plant tissues, with concentrations in edible plant parts between 0.02 and 2.58 mg kg^−1^. Our Cd concentrations in the plants went up to 0.68 mg kg^−1^ and was found in peppers. Therefore, Cd was not as easily translocated into the aboveground plant parts, which could have been due to soil type and plant species/cultivars. The Cd values in our study and the study by Králová et al.^31^ in majority of cases surpassed both the limits set by the Ministry of Health in the Czech Republic^9^ and the European directive^27^. In the case of Zn, high concentrations found by Králová et al.^31^ ranged between 21 and 228 mg kg^−1^ were similar to the results from this experiment (11.83 to 153 mg kg^−1^), were concluded as not phtotoxic. Concentrations of Pb ranged between 0.04 and 1.03 mg kg^−1^ in the study by Králová et al.^31^, while in our results, Pb concentrations were significantly higher (0.43 to 4.42 mg kg^−1^). Our results exceeded the MAQ and the European directive, which states the limit of Pb in foodstuff as 0.10 mg kg^−1^. Therefore, PTE concentrations in plants are highly influenced by the plant species and the soil physio-chemical properties. Despite the low plant availability of PTE, concentrations in plants studied in this experiment still exceeded the guideline values set for edible plants, as shown in Table 3.

Potentially toxic elements in soil can transfer to humans in a number of ways, including the direct consumption of contaminated soil particles with unwashed vegetables, on unwashed hands, through soil ingestion by children, infants, and pets, by inhalation of dust, or through uptake into edible vegetables^32^. A further exposure to soil PTE is by its inadvertent transport to the inside of houses from the garden; Laidlaw et al.^33^ showed that the source of interior Pb dust was primarily from soil in two out of three houses. Izquierdo et al.^34^ performed a comprehensive risk assessment for PTE bioaccessible in urban gardens. Their conclusions highlighted a combined exposure for children; soil ingestion due to play, and consumption of vegetables grown on contaminated soil. Drahota et al.^3^ found health risks, especially related to As, associated with ingestion of mine waste materials and contaminated urban soils. In several localities surrounding KH, mine waste slags were re-cultivated into gardens and fields^35^, therefore posing a risk to humans.

Soil PTE levels vary and are difficult to predict in city vegetable gardens due to the heterogeneous nature of urban pollution and past land uses. Nonetheless, many affordable and feasible (for households) remediation techniques exist that can help decrease the plant available fractions. Such remediation techniques involve the incorporation of clays, compost, biochar, clean top-soils, or by providing a crop-cover, and by growing ornamental plants rather than edible ones. Such remediation techniques have been considerably studied with promising results^35–41^. However, when implementing amendment measures, several factors must be taken into account. Soil properties (eg. pH, soil organic matter, Cox, etc.) as well as the type of contamination and the main contaminants present are the most important factors. Implementing amendment for As contamination widely differ from amendments that would work for Zn or Pb, for example.

The plants with the highest overall PTE concentrations were peppers, potato tubers, tomatoes and cucumbers, therefore the gardeners are recommended to avoid planting these plant species in their gardens or to use different cultivars that could possibly accumulate less PTE in the edible plant parts. The plant with the lowest uptake of As and Cd into the edible plant parts were apples, therefore, planting fruit trees rather than vegetables, could be a solution. While growing of ornamental plants instead of edible ones is a tactical way to combat plant to human transfer of risk elements in the garden soils surround KH, another possibility is the plantation of trees. Trees have the ability to retain risk elements bound in soils, albeit the uptake ability of trees can be relatively low and depends on the level of soil contamination^42,43^. The chosen amendment would differ greatly from garden to garden depending on the plants cultivars, the soil type and the highest PTE present in the soils and plants.

## Conclusion

There is a risk of transfer of As (and other PTE) from soil, close to a historical mining site, to self-grown vegetables via root systems. Growing vegetables and fruits in the contaminated soils present certain risks to consumers and therefore, certain measures must be taken to decrease the high concentrations of PTE that can be taken up by the edible fruits and vegetables.

Out of the four studied PTE, As was the element that was present in excessive quantities in garden soils, with the lowest concentration in the soil surpassing the SBL. Despite the low 1 M NH_4_NO_3_ extractable (plant available) As, it was still the most plant available element studied. Concentrations of PTE in edible plant parts exceeded allowable quantities (0.5 mg As kg^−1^; 0.05–0.1 mg Cd kg^−1^; 0.1 mg Pb kg^−1^; 10–25 mg Zn kg^−1^) set by the Czech Ministry of Health, with peppers, potato tubers, tomatoes, and cucumbers accumulating the highest PTE concentrations in their tissues. Based on the observed soil and plant PTE concentrations, vegetable owners are encouraged to take certain measures to mitigate the contamination. Incorporating clean topsoil, clays, compost, Fe-oxides or biochar to the plots can reduce the relative PTE concentration in soil, however, managing soils with multiple risk elements can be problematic as what could work for As, could potentially have a different effect on Cd. Grass-seeding on unused plots or maintaining a crop cover for as much of the year as possible will reduce contaminated dust migration. Furthermore, in case of soil-to-plant transfer of mobile risk elements, such as As, growers should consider growing ornamental plants or trees instead of edible plants in the worst affected soils. A possible solution to prevent further contamination of land through wind or water erosion from unconfined deposits would be through land reclamation, such as reforesting the area.

## Data Availability

The data that support the findings of this study are openly available on request. Please contact the corresponding author (vejvodova@af.czu.cz) for further information.
